# Additional notes on the morphology and molecular data of the Kikuyu root-knot nematode, *Meloidogyne kikuyensis* (Nematoda: Meloidogynidae)

**DOI:** 10.21307/jofnem-2020-067

**Published:** 2020-08-12

**Authors:** J. D. Eisenback, P. Vieira

**Affiliations:** Professor and Research Scientist, respectively School of Plant and Environmental Science, Virginia Tech, Blacksburg, VA, 24061

**Keywords:** Mitochondrial DNA, SEM, Sugarcane, Systematics, Taxonomy

## Abstract

Females, males, and second-stage juveniles of *Meloidogyne kikuyensis* were examined by light and scanning electron microscopy. The morphology of *M*. *kikuyensis* was typical for species of the genus in general, but differed in several characters, appearing to be in a more primitive state. The head morphology of males and second-stage juveniles of most species of root-knot nematode is made up of a large labial disk surrounded by the fused pairs of the sub-dorsal and sub-ventral lips, but in *M*. *kikuyensis*, the labial disk is surrounded by six distinct lips. Second-stage juveniles appear to develop similarly to that of other members of the genus. The division of the egg seems to be quite different from typical species in that two small, highly refractive cells, are set-aside early in embryogenesis. Elucidation of the mitochondrial nucleotide sequence for the cytochrome oxidase subunit II and the large subunit of the ribosomal RNA gene (COII-16S rRNA) and the ITS1 region implicated *M. kikuyensis* is in a basal position when compared to other species of the genus.

The roots of sugarcane (*Saccharum officinarum* L.) in Zululand, South Africa were heavily infested with small, nodule-like galls. Upon closer examination, *Meloidogyne kikuyensis* De Grisse, 1961 was identified infecting the roots (Eisenback and Spaull, 1988). This species was originally described parasitizing kikuyu grass (*Pennisetum clandestinum* Höchst.) in Muguga, Kenya by De Grisse (1960) who stated that the galls ‘resembled offset leguminous nodules to some extent.’ Recently, details of the nodule-like gall were elucidated by light, scanning, and transmission electron microscopy (Eisenback and Dodge, 2012; Dodge, 2014). The galls induced by *M. kikuyensis* are unique and more complex than those caused by most root-knot nematode species. The vascular tissues that supply the giant cells with nutrients occur at a right angle to the vascular cylinder in the main root. Unlike most species of root-knot nematodes, feeding cells of *M. kikuyensis* appear to be formed by the dissolution of cell walls that contribute to the makeup of the enlarged giant cells (Dodge, 2014).

Cytological investigations of this species by Triantaphyllou (1990) showed that *M. kikuyensis* has only seven chromosomes, like that of *M. spartinae* Rau and Fassuliotis, 1965, and they are at least twice as big as those of all other *Meloidogyne* species examined. The chromosome number of most species of root-knot nematodes is *n* = 13-19, which caused Triantaphyllou (1990) to speculate that *M. kikuyensis* is a primitive species of *Meloidogyne* because of the large size and low number of chromosomes.

Studies on the morphology of this species were initiated to confirm our preliminary identification, to contribute additional information about the morphology of this species, and to complete a promise made (Triantaphyllou, 1990). Because of the nodule-like morphology of the gall, the complex and unique feeding site, and the unusual chromosome number, size, and behavior, the morphology and molecular biology of *M. kikuyensis* was examined in detail to evaluate it as a putative primitive species within the root-knot nematodes and to determine if it, indeed, belongs to this genus.

## Materials and methods

*Meloidogyne kikuyensis* was obtained from a sugarcane field in Zululand, South Africa by V. W. Spaull in 1984. The population was maintained on sugarcane under normal greenhouse conditions (18-24°C). Males, females, and second-stage juveniles were obtained from numerous and characteristic nodule-like galls that formed on infected roots.

Specimens were prepared for scanning electron microscopy (SEM) by sequential fixation and freeze-drying (Eisenback, 1987). Stylets were extracted in 45% lactic acid as previously described (Eisenback et al., 1980). Perineal patterns were made as described by Hartman and Sasser (1985) and observed according to Eisenback (2010). Whole specimens prepared for light microscopy (LM) were mounted on 4% water agar blocks (Eisenback, 2012). They were photographed with a Nikon D300 DSLR camera attached to a Dialux 22 Leitz microscope.

Cowpea (*Vigna unguiculata* (L.) Walp.) and yellow foxtail (*Setaria glauca* (L.) Beauv.) were tested as additional hosts of *M. kikuyensis* by inoculating one plant with 3,000 eggs each and maintained in the greenhouse for 45 days at a temperature of 18 to 24°C. Only one plant was used because eggs were always difficult to find in large numbers.

Sequencing of the fragment between mitochondrial cytochrome oxidase subunit II gene and the large (16S) subunit of the ribosomal RNA gene (COII-16S) was amplified using the forward C2F3 (5´-GGT CAA TGT TCA GAA ATT TGT GG-3´) and reverse 1108 (5´-TAC CTT TGA CCA ATC ACG CT-3´) primers (Powers and Sanders, 1993). The following primers were used for the amplification of the ITS1 region, including partial sequences of the 18S and 5.8S rRNA genes: 5´-TTG ATT ACG TCC CTG CCC TTT-3´ (Vrain et al., 1992) and 5´-ACG AGC CGA GTG ATC CAC CG-3´ (Cherry et al., 1997). The obtained sequences for *M. kikuyensis* were deposited in GenBank with the accession numbers MN631057 and MN634198, respectively. Multiple sequence alignment of the COII-16S rRNA region and the ITS1 region for a set of *Meloidogyne* species retrieved from GenBank were conducted using ClustalX. Their phylogenetic relationships were estimated using maximum likelihood (ML) analysis using the HKY model implemented at CLC Workbench v. 8. The robustness of ML analysis was inferred using 1,000 bootstrap replicates. As outgroup *taxa*, the corresponding sequences of COII-16S rRNA of *Radopholus similis* and ITS1 of *Hirschmanniella mucronata* were used.

## Results

### Female (Figs. 1, 2)

**Figure 1:**
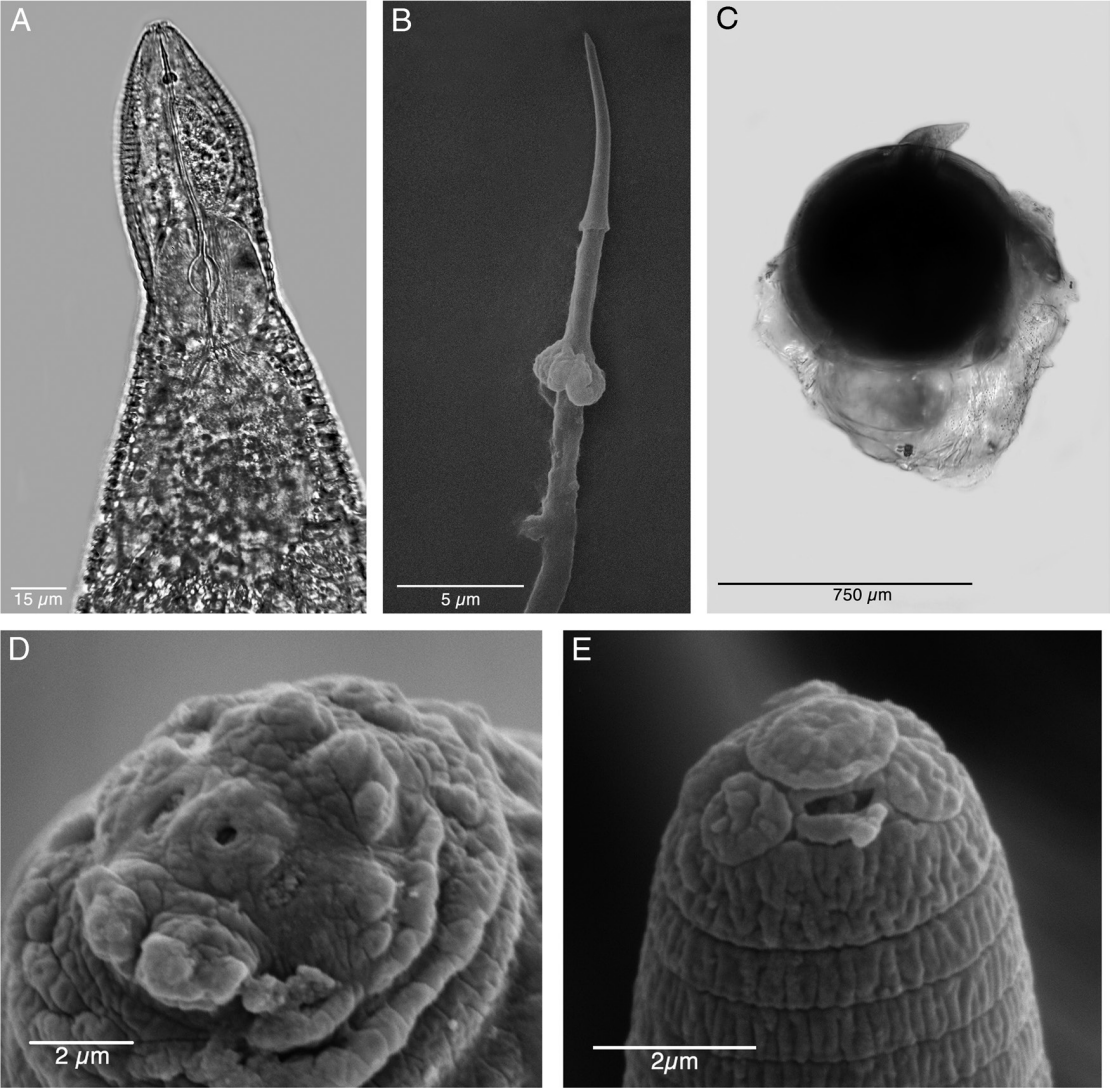
Light (LM) and scanning electron micrographs (SEM) of females of *Meloidogyne kikuyensis* De Grisse, 1961. A: LM of the anterior end showing the stylet and esophagus. B: SEM of an extracted stylet. C: LM of a whole specimen with attached gelatinous matrix secreted by the six rectal gland cells. D: SEM of female anterior end. E: SEM of the anterior end of a second-stage juvenile.

**Figure 2:**
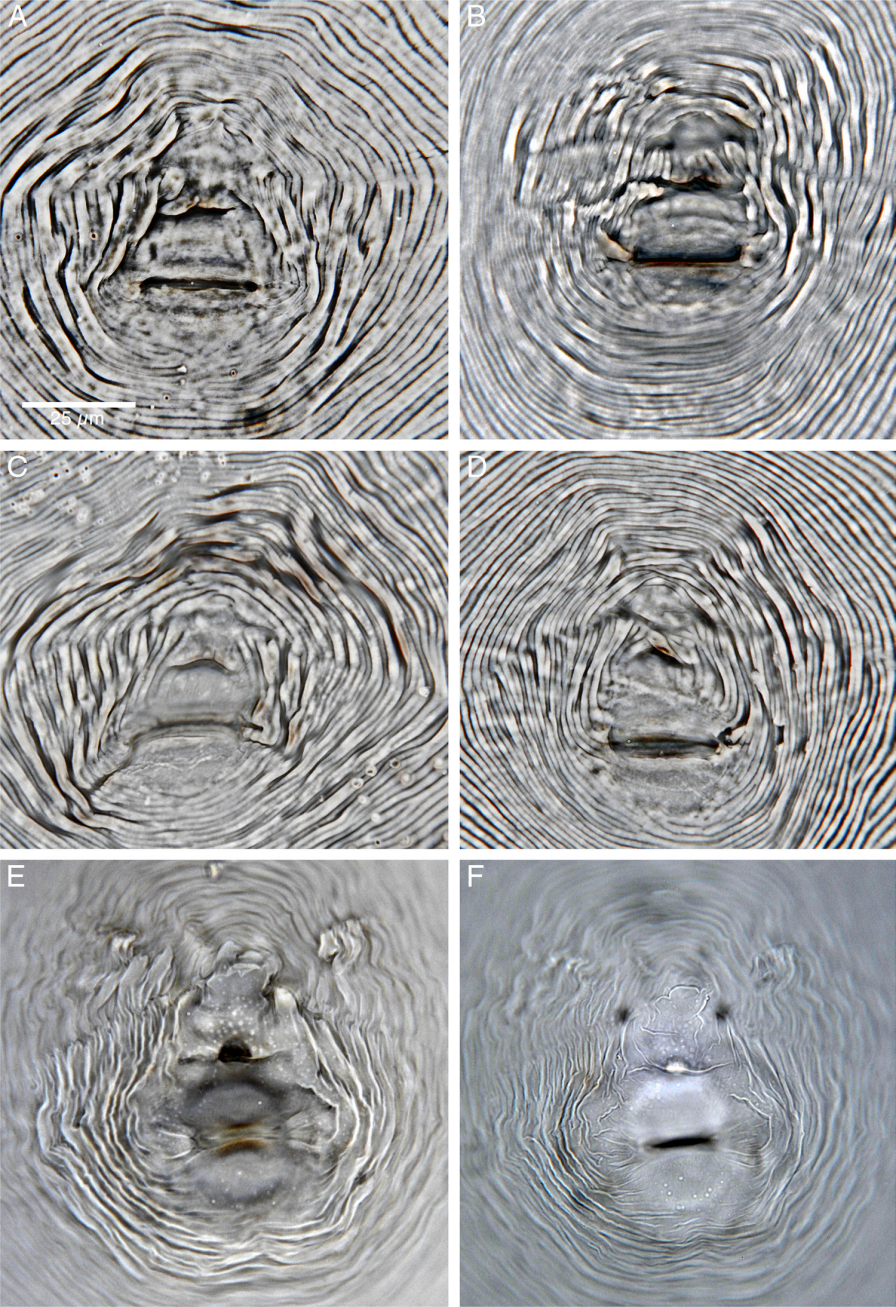
Light micrographs (LM) of perineal patterns of *Meloidogyne kikuyensis* De Grisse, 1961. A-E: LM with polarizing rings in the condenser (Eisenback, 2010). F: LM utilizing typical brightfield microscopy.

The body is nearly round to sub-spherical in shape. The stylet is slender and the cone has a slight dorsal curve; the knobs are divided anteriorly and irregular in their surface morphology and they slope posteriorly. The dorsal esophageal gland orifice (DGO) is 3.5 to 5 (4.0) µm from stylet base. The median bulb is large, rounded, and is lined with triradiate crescentic thickenings in the lumen. The esophagus contains one large dorsal gland lobe, two smaller sub-ventral gland lobes, and two rounded esophageal-intestinal cells. The morphology of the entire esophageal structure is typical for members of *Meloidogyne*.

The excretory pore occurs usually near the level of the DGO. The ovary is followed by the spermatheca and oviduct. The spermatheca is made up of 28 to 34 large rounded cells with undulating borders, and the oviduct contains six to eight cells. The anatomy of the ovary is not normal for the genus *Meloidogyne*, but is similar in structure to several other early branching species, such as *M. ichinoei* Arachi, 1992, *M. africana* Whitehead, 1968, and *M. mali* Itoh et al., 1969 (Chizhov, 1981; Triantaphyllou, 1990; Toon et al., 2017).

The perineal pattern of *M. kikuyensis* has a high, square dorsal arch. The striae are coarse, and the tail tip is free of striae. Distinct lateral lines are absent; however, the dorsal and ventral lines meet and form slight impressions of lateral lines. Phasmids are indistinct. Striae surrounding the tail remnant continue ventrally and turn toward the vulva, forming characteristic cheek-like formations. In total, 10 to 12 horizontal striae may occur between the anus and the tail remnant, or it may be free of striae. Overall, the perianal pattern contains all of the markings and structures associated with a typical species within genus.

### Male (Figs. 3-5)

**Figure 3:**
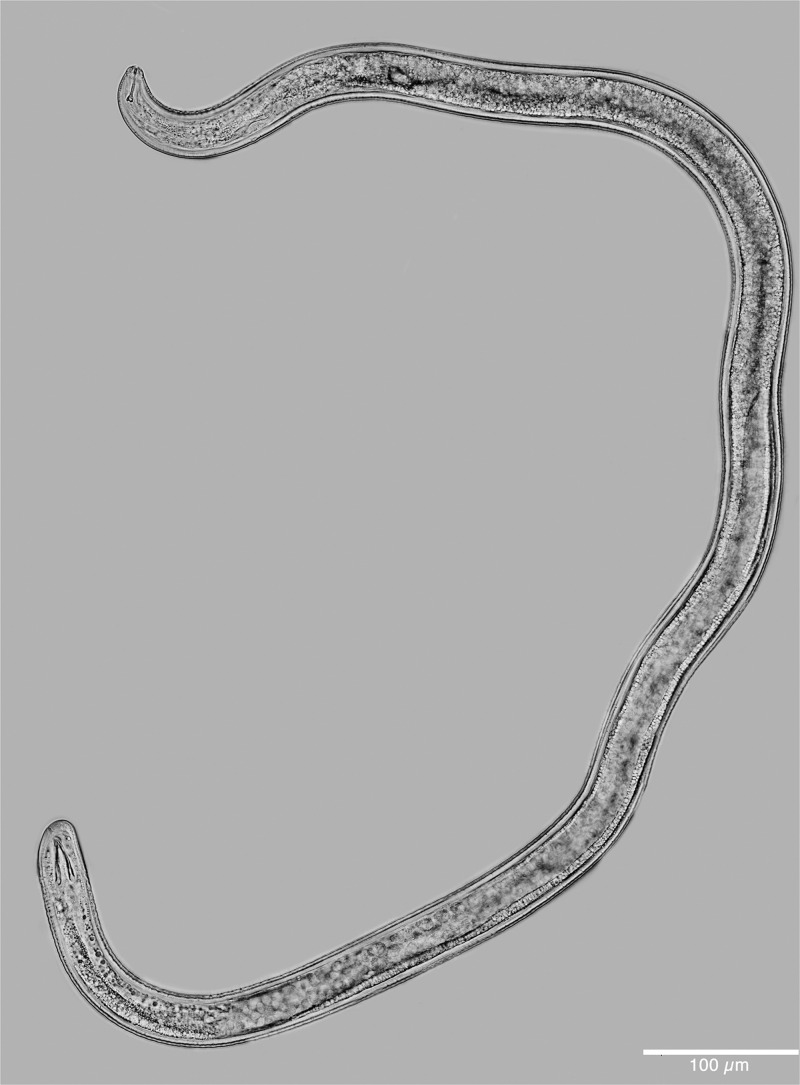
Light micrograph of a whole specimen of a male of *Meloidogyne kikuyensis* De Grisse, 1961.

**Figure 4:**
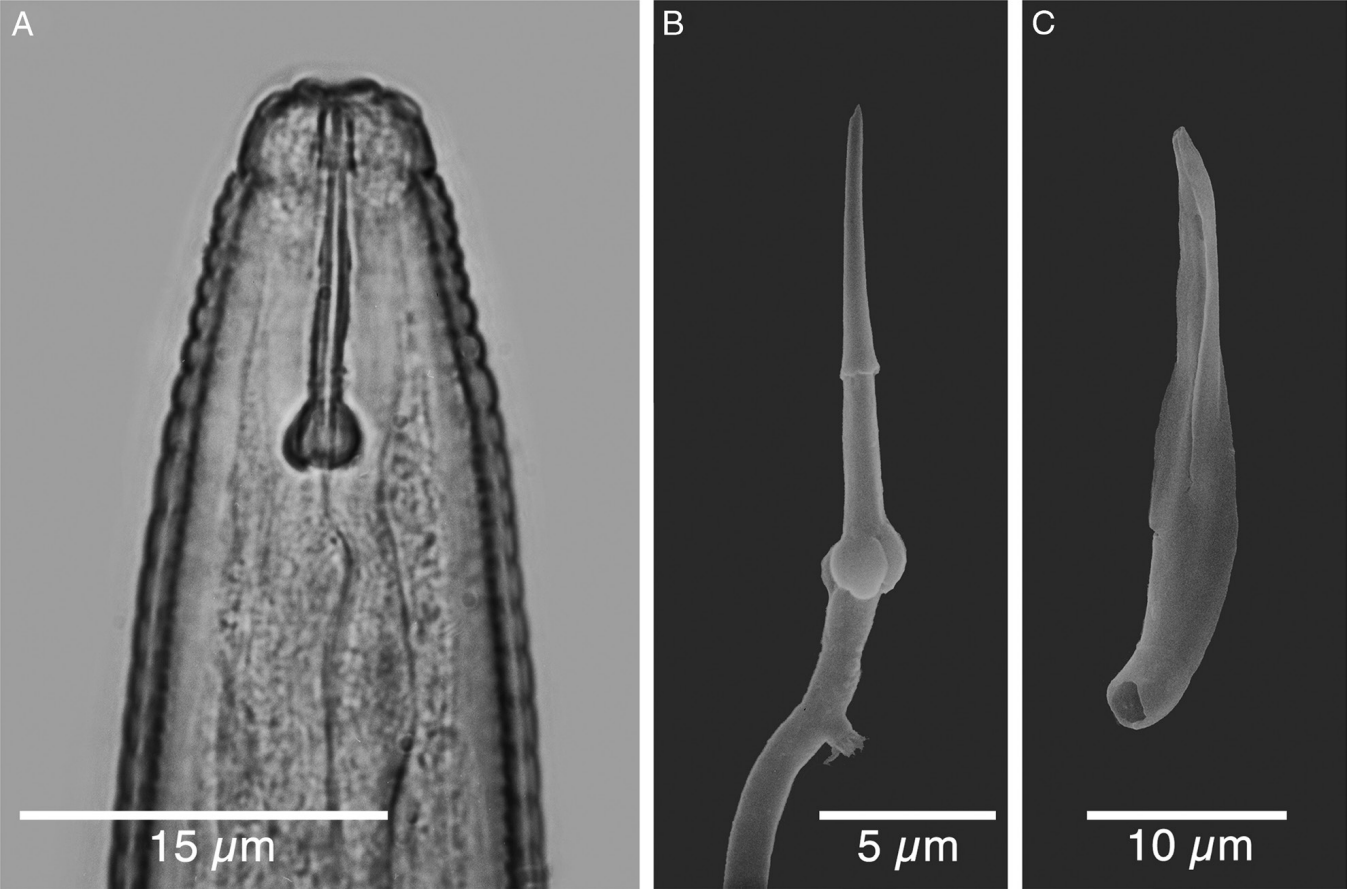
Light (LM) and scanning electron micrographs (SEM) of males of *Meloidogyne kikuyensis* De Grisse, 1961. A: Anterior end. B: SEM of an excised stylet. C: SEM of an excised spicule.

**Figure 5:**
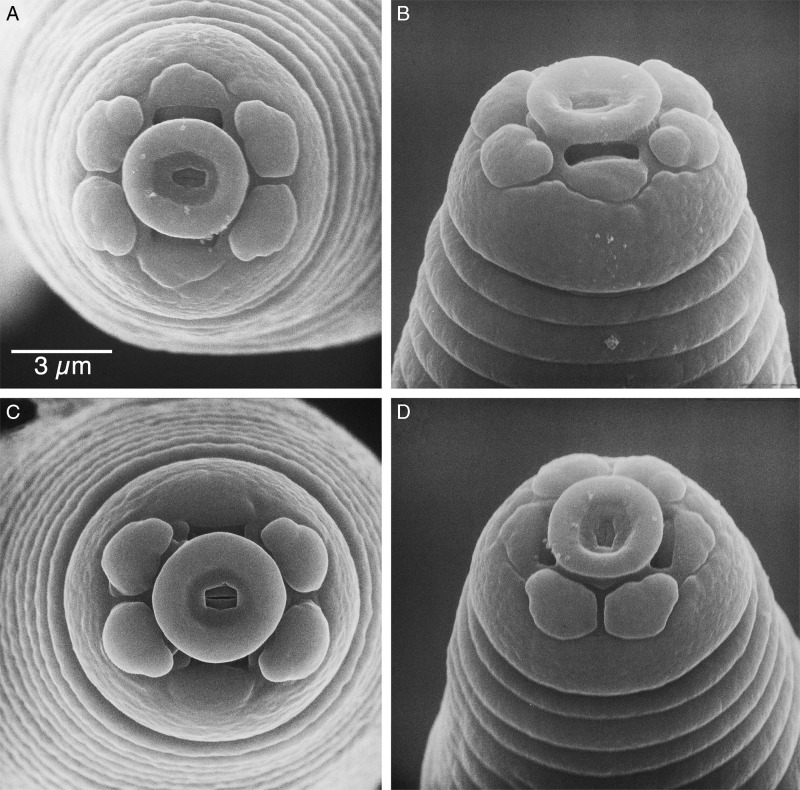
Scanning electron micrographs of the anterior end of males of *Meloidogyne kikuyensis* De Grisse, 1961. A: Face view showing a male with a distinct labial disk and six lips. B: Lateral view. C: Face view showing a male with just four lips. D: Medial view showing a male with six distinct lips.

In the light microscope, the male is very long and tapered in the head region and has a rounded tail that is twisted 90° in relation to the rest of the body. The dome-shaped head cap is set-off from the regular body annulation. The labial disk is distinctly concave and separated from the lips. SEM revealed that some males contain a full complement of six smooth, rounded lips, whereas in others, the lateral lips are completely fused with the head region. Cepahlic sensilla are not visible on the medial lip pairs. The elongated hexagonal prestoma is marked by six pit-like inner labial sensilla. The stoma is slit-like. The head annule is usually smooth and not marked with additional annulations.

The morphology of the stylet is unique for the species, but spicule morphology is similar to that of other species in the genus. The stylet is nearly straight and the knobs are rounded, sometimes indented anteriorly, and the surface is somewhat irregular. They slope backwards. The DGO is long (4.5-6) 5 µm. The pharyngeal glands are variable in shape and size, but usually have one nucleated dorsal gland lobe and two nucleated sub-ventral gland lobes.

The testis is always single and not reflexed. The vas deferens is full of spermatocytes and large, rounded sperm. The morphology of the testis and secondary sexual structures are typical for the genus *Meloidogyne*.

### Second-stage juvenile (Figs. 1, 6-8)

**Figure 6:**
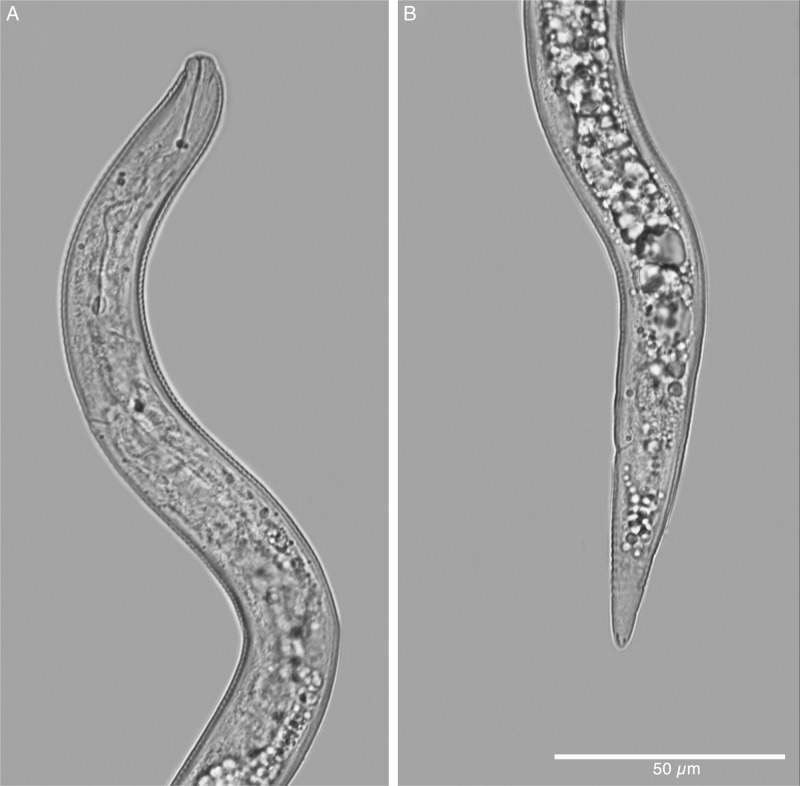
Light micrograph of a second-stage juvenile of *Meloidogyne kikuyensis* De Grisse, 1961. A: Anterior end. B: Posterior end.

**Figure 7:**
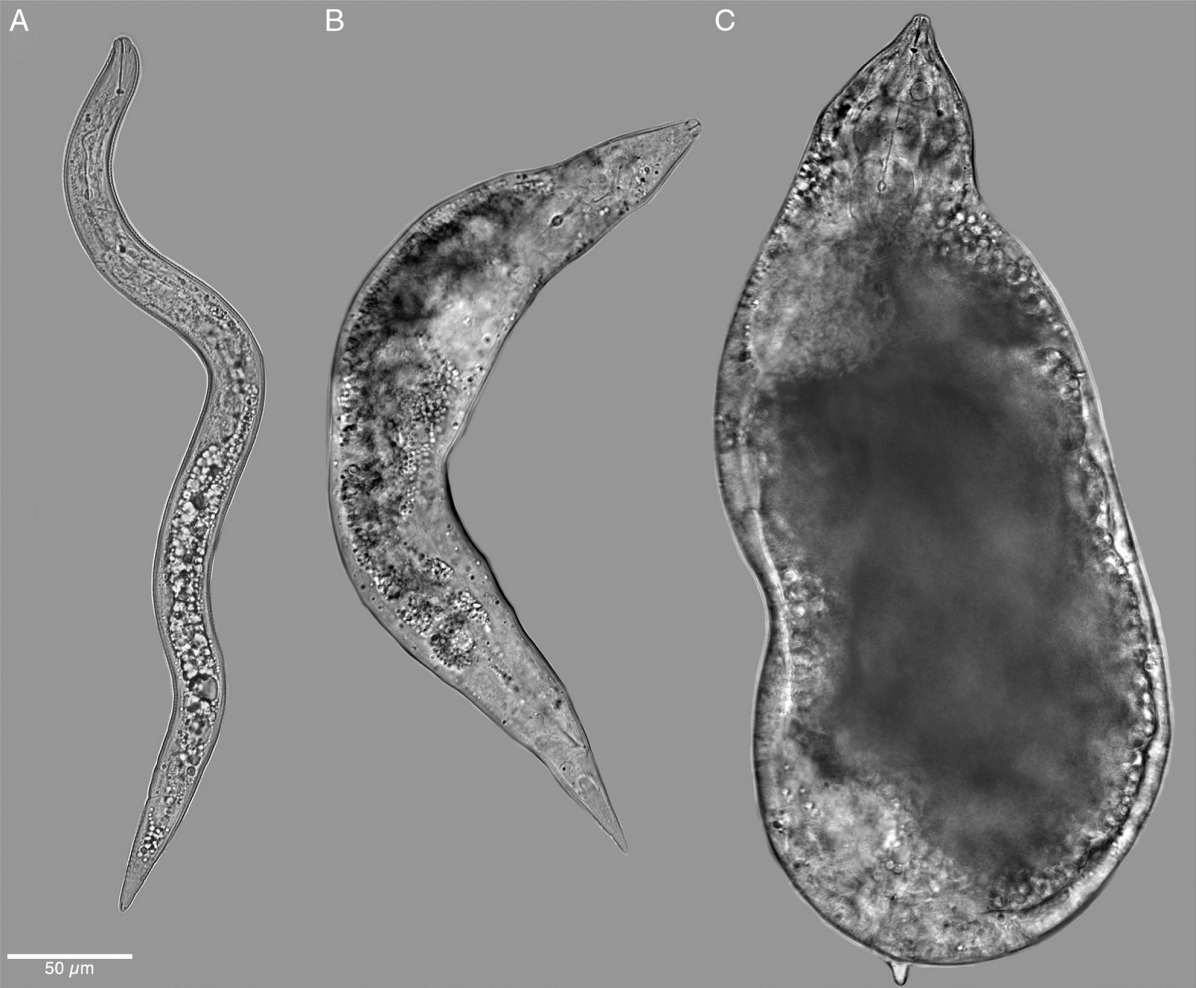
Light micrograph of second-stage juveniles of *Meloidogyne kikuyensis* De Grisse, 1961. A: Pre-infective migratory second-stage juvenile. B: Slightly swollen post-infective sedentary second-stage juvenile. C: Swollen post-infective second-stage juvenile.

**Figure 8:**
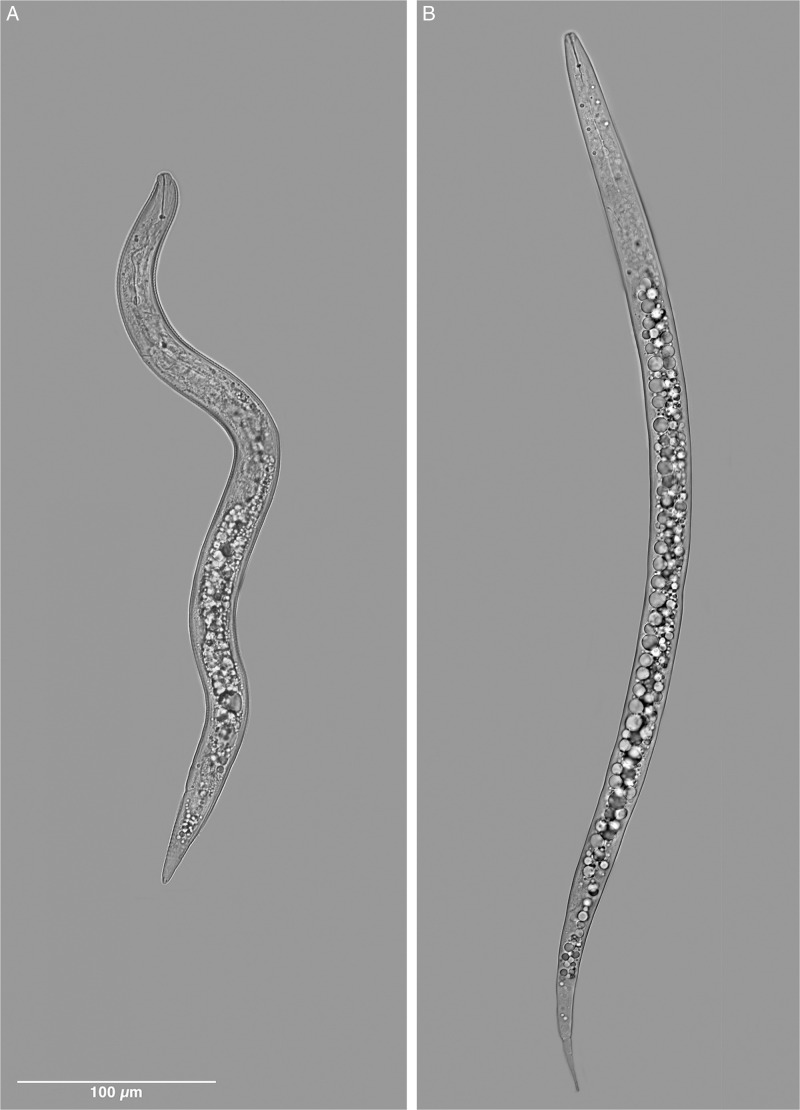
Light micrographs comparing the second-stage juvenile of *Meloidogyne kikuyensis* De Grisse, 1961 with that of a typical root-knot species.

Second-stage juveniles are fusiform. The head annule is set-off from the rest of the body. Like that of the male, the distinct labial disk is surrounded by six individual lips, two sub-dural, two sub-ventral, and two lateral. The stylet is long (12-15 µm) and the knobs are small and rounded. Development of the vermiform pre-infective stage to that of the swollen parasitic stage appears to be typical for the root-knot nematodes. Compared to other species of root-knot, the second-stage juveniles are short (290-360 µm) and thick in diameter (14-18 µm), and the tail is likewise short (45-51 µm).

### Egg (Fig. 9)

The division of the egg seems to be quite different from typical species in that two small, highly refractive cells are set-aside early in embryogenesis and appear to surround the developing embryo. Although these cells were observed in a number of eggs, not enough specimens were available to follow these cells through the development of the egg.

**Figure 9:**
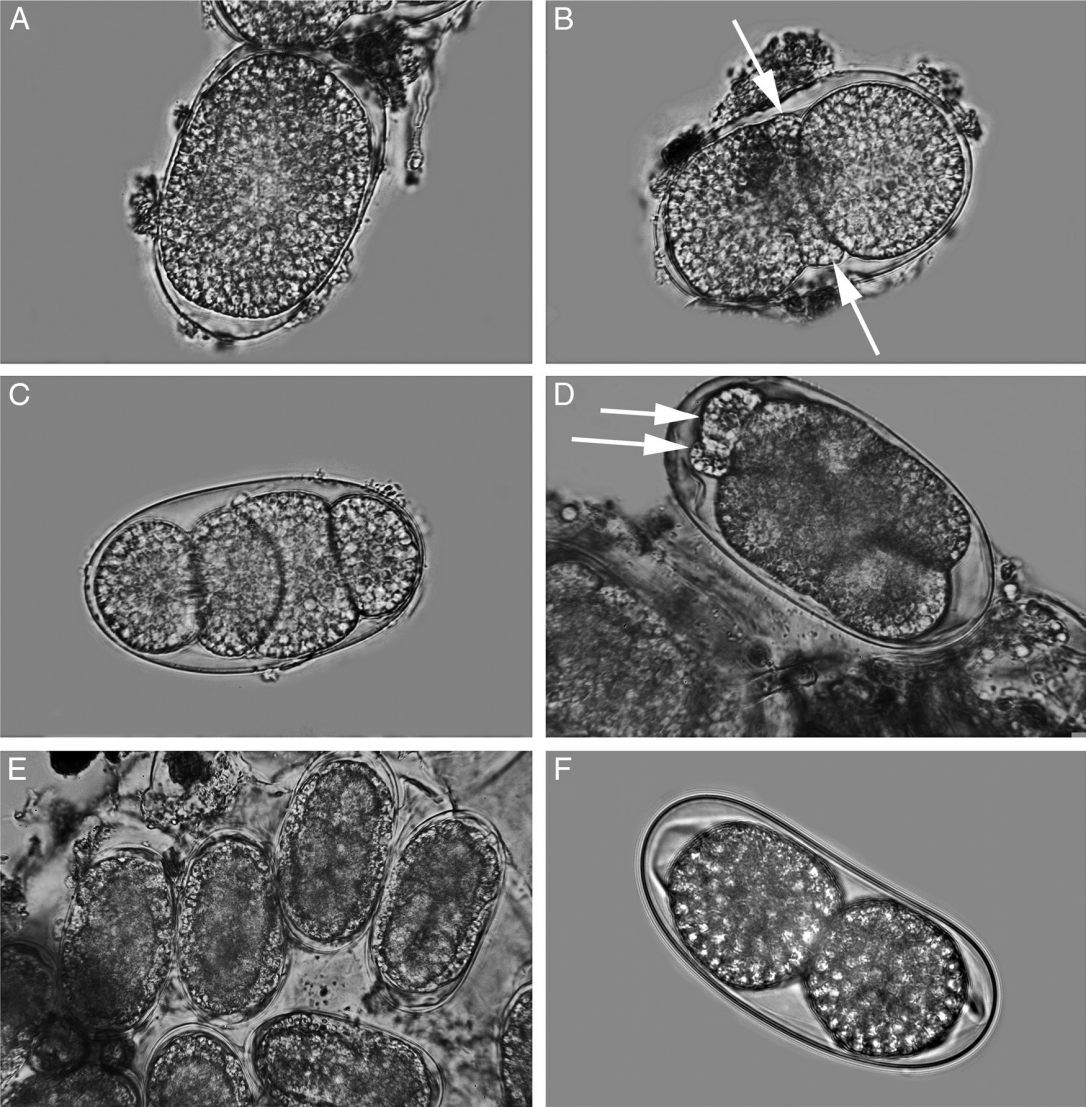
Light micrograph of eggs of *Meloidogyne kikuyensis* De Grisse, 1961. A: Single-cell stage. B: Two-cell stage with small, highly refractive polar cells (arrows). C: Four-cell stage. D: Eight-cell stage showing two small, highly refractive polar cells (arrows). E, F: Multi-cell stage of several eggs showing a ring of refractive cells (arrows) surrounding the developing embryo.

## Molecular relationships

**Figure 10:**
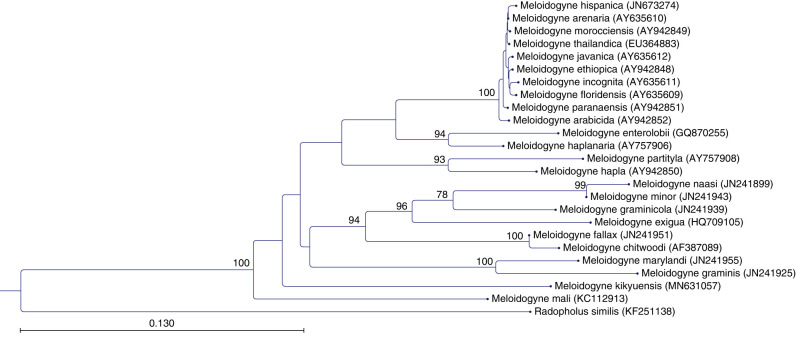
Phylogenetic relationships based on the COII-16S sequences of *Meloidogyne kikuyensis* De Grisse, 1961 and other *Meloidogyne* species. The phylogenetic tree was deduced by maximum likelihood (ML) analysis with the HKY model and 1,000 bootstrap replicates (only values of bootstrap above 60% are shown). The COII-16S sequence of *Radopholus similis* was used as an outgroup.

**Figure 11:**
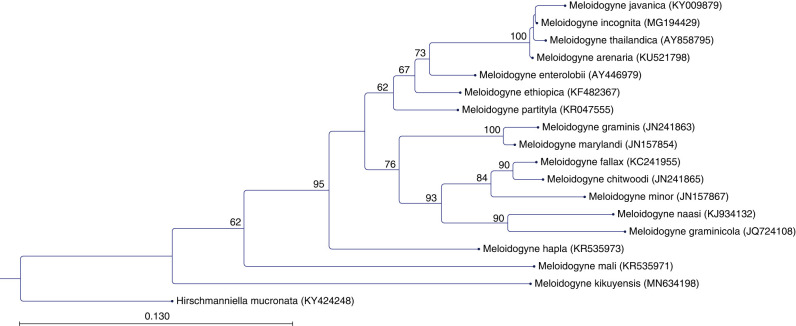
Phylogenetic relationships based on the ITS1 sequences of *Meloidogyne kikuyensis* De Grisse, 1961 and other *Meloidogyne* species. The phylogenetic tree was deduced by maximum likelihood (ML) analysis with the HKY model and 1,000 bootstrap replicates (only values of bootstrap above 60% are shown). The ITS1 sequence of *Hirschmanniella mucronata* was used as an outgroup.

The amplification of the COII-16S rRNA yielded a fragment of 544 bp for *M. kikuyensis* (MN631057), following other *Meloidogyne* species belonging to the smallest size class, which lack an AT-rich region of the corresponding mitochondrial region. However, the COII-16S rRNA sequence of *M. kikuyensis* is distinct when compared to other available sequences. The resulting phylogenetic tree (Fig. 10) using the corresponding COII-16S rRNA sequences of *M. kikuyensis* together with other *Meloidogyne* species places *M. kikuyensis* separately from the other species, which highlights its differences among other species within the genus. For the ITS1 region yielded a fragment of 500 bp, including both partial regions of the 18S rRNA region and the 5.8S region (MN634198). The inferred ML trees revealed a similar topology as the COII-16S tree, showing a more basal position of *M. kikuyensis* with other *Meloidogyne* sequences retrieved from NCBI (Fig. 11).

## Discussion

The morphology of the female, male, and second-stage juvenile of *M. kikuyensis* is overall typical for the genus; however, the labial disk and lips are distinct and separate from each other, unlike most other species. Females have a characteristic stylet morphology in the shape and fine details of the knobs. The overall shape and details of the perineal pattern are different from other species. The cheek-like striae at the edges of the vulval slit may have been emphasized too much. Although these striae occur in many specimens, they are not always present. The anatomy of the digestive and reproductive systems is typical for the genus, but the spermatheca of the female is more primitive. It is made up of 28 to 34 large rounded cells with undulating borders, and the oviduct contains six to eight cells. The anatomy of the ovary is similar in structure to several other early branching species (*M. ichinoei* *M. africana* and *M. mali* (Chizhov, 1981; Triantaphyllou, 1990; Toon et al., 2017).

Likewise male head morphology of *M. kikuyensis* is very different from that of the four most common species and all others species that have been examined by SEM. They have a unique conically depressed labial disk surrounded by six distinct lateral, sub-ventral, and sub-dorsal lip pairs. Lateral lips are present in most males, but absent in some. The occurrence of a full complement of six lips and three esophageal glands indicates that *M*. *kikuyensis* may be a primitive species within the genus.

The development of the egg seems to be unique with early divisions separating two highly refractive cells that persist during embryogenesis and appear to further divide and completely surround the developing embryo.

The limited test for additional hosts of this nematode was not successful. No galls were found on cowpea, and a few galls developed on yellow foxtail, but the females within the galls did not produce any eggs. The failure to reproduce on cowpea questions the likelihood of this plant as a host (Olowe, 2009). Coffee (*Coffea arabica* L.) has also been reported as a host, but details about the host-parasite relationship are scant (Swai, 1981; Bridge, 1984; Campos and Villain, 2005), and Whitehead reported that coffee was resistant to this species (Whitehead, 1969).

Onkendi et al. (2014) reported *M. kikuyensis* in Kenya, South Africa, and Tanzania on Kikuyu grass *Pennisetum clandestinum* Hochst. ex Chiov. and sugarcane (De Grisse, 1960; Kleynhans, 1991). The first report of *M. kikuyensis* in Italy on carnation (*Dianthus caryophyllus* L.) was probably a misidentification because it was based primarily on a perineal pattern of suspicious morphololgy (Scognamiglio and Varma, 1982).

Sequence alignment of *M. kikuyensis* with other *Meloidogyne* species available at NCBI showed that *M. kikuyensis* belongs to the group of *Meloidogyne* species that have a shorter COII-16S fragment. The corresponding phylogenetic analyses suggest a clear separation of *M*. *kikuyensis* from those species having a shorter fragment, including *M. mali*. Both COII-16S and ITS1 trees suggest a basal position of *M. kikuyensis* and other species of the genus.

We agree with Triantaphyllou (1984) and Toon et al. (2017) that *M. kikuyensis* can be considered a primitive member of the genus because of the head morphology of females, males, and second-stage juveniles; the small chromosome number (*n* = 7); the reduced morphology of the spermatheca (Chizhov, 1981); and the molecular relationship inferred with other *Meloidogyne* species. Unfortunately, molecular data from this species was not available for the recent analysis of the phylogeny of the genus by Álvarez-Ortega et al. (2019). In spite of a lengthy search for additional material of *M. kikuyensis,* we were unable to find a suitable source that would allow us to sequence the D2 to D3 region of the 28s RNA gene and add more directly to the understanding of the phylogeny of *Meloidogyne*.
